# Multi-Objective Association Detection of Farmland Obstacles Based on Information Fusion of Millimeter Wave Radar and Camera

**DOI:** 10.3390/s23010230

**Published:** 2022-12-26

**Authors:** Pengfei Lv, Bingqing Wang, Feng Cheng, Jinlin Xue

**Affiliations:** 1 College of Engineering, Nanjing Agricultural University, Nanjing 210031, China; 2 Jiangsu Agricultural Machinery Information Center, Nanjing 210031, China

**Keywords:** information fusion, millimeter wave radar, camera, farmland obstacle, target detection

## Abstract

In order to remedy the defects of single sensor in robustness, accuracy, and redundancy of target detection, this paper proposed a method for detecting obstacles in farmland based on the information fusion of a millimeter wave (mmWave) radar and a camera. Combining the advantages of the mmWave radar in range and speed measurement and the camera in type identification and lateral localization, a decision-level fusion algorithm was designed for the mmWave radar and camera information, and the global nearest neighbor method was used for data association. Then, the effective target sequences of the mmWave radar and the camera with successful data association were weighted to output, and the output included more accurate target orientation, longitudinal speed, and category. For the unassociated sequences, they were tracked as new targets by using the extended Kalman filter algorithm and were processed and output during the effective life cycle. Lastly, an experimental platform based on a tractor was built to verify the effectiveness of the proposed association detection method. The obstacle detection test was conducted under the ROS environment after solving the external parameters of the mmWave radar and the internal and external parameters of the camera. The test results show that the correct detection rate of obstacles reaches 86.18%, which is higher than that of a single camera with 62.47%. Furthermore, through the contrast experiment of the sensor fusion algorithms, the detection accuracy of the decision level fusion algorithm was 95.19%, which was higher than 4.38% and 6.63% compared with feature level and data level fusion, respectively.

## 1. Introduction

The unmanned technology of agricultural machinery is an important development direction [1,2]. The unmanned agricultural machinery should have a certain perception ability to carry out autonomous operation and behavior decision [3,4], because there are inevitably some dynamic and static obstacles on their travel route, such as people, trees, houses, sheep, wire poles, and other agricultural machinery. If these obstacles cannot be detected and identified, it will cause serious losses once the unmanned agricultural machinery collides with the obstacles.

In agricultural fields, single or multiple sensors such as LIDAR, vision sensors, structured light sensors, ultrasonic sensors are usually used for obstacle detection [5,6,7,8]. In [9], the authors proposed an improved YOLOv5s algorithm for vision detection of farmland obstacles, which can improve detection precision and speed up the real-time detection.. In [10], the author introduced data acquisition, processing technology and post-processing technology to demonstrate the ability of detecting partially obscured targets from foliage with mmWave radar. In fact, the information obtained by a single sensor is poorly reliable, because it can lead to wrong or large error decisions for unmanned agriculture machinery in case of sensor failure as well as mismeasurement in certain scenarios. Therefore, information fusion of multiple sensors such as Lidar, camera, and other sensors are generally applied for a more comprehensive description of farmland obstacles [11,12,13,14].

In fact, the mmWave radar and monocular camera are inexpensive. In addition, mmWave radar has better performance in range and speed measurement, while monocular camera has greater advantages in lateral positioning and type recognition [15]. Therefore, the combination of the two can fully exploit their strengths and complement each other’s information to achieve maximum benefits in farmland obstacles detection.

The advantages of the information fusion between the mmWave radar and camera are threefold. 

**Figure 1:**
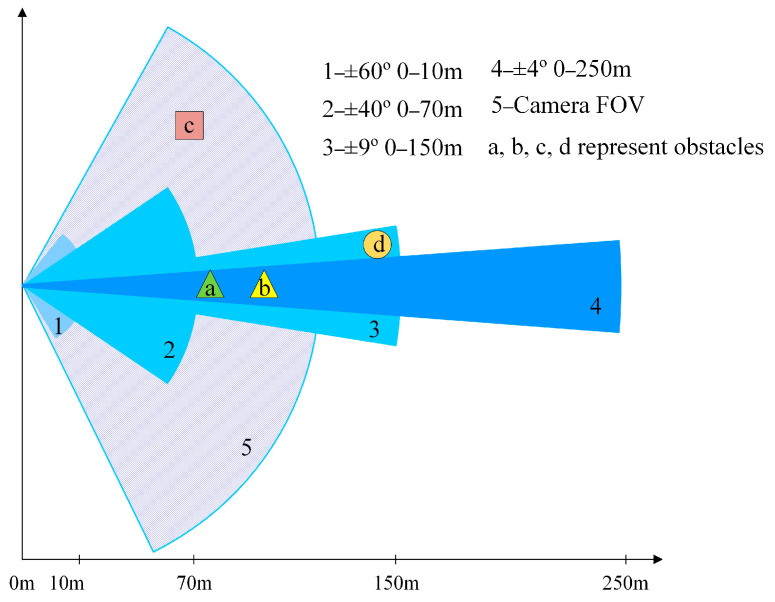
Visual field angle of fusion of single sensor and multi-sensor.

(1) The range of perception is expanded. As shown in Figure 1, the mmWave radar and the camera perform obstacle sensing separately, but after the decision-level fusion processed in this paper, the unmatched targets (e.g., obstacles c and d in Figure 1) are retained in the effective life cycle. Therefore, the detection advantages of both sensors in both longitudinal and lateral directions are complementary.

(2) The information integrity of the target is improved. The longitudinal velocity information and target type information are added for successfully matched targets, and the lateral and longitudinal distances are weighted to take full advantage of the camera at the lateral direction and the mmWave radar at the longitudinal direction. 

(3) The missing detection of the camera caused by occlusion is solved. As shown in Figure 1, object b is blocked by object a and cannot be detected by the camera, while the electromagnetic waves of the mmWave radar have penetrating properties and can detect the blocked object. 

Therefore, the motivation of this paper is to propose a method for detecting obstacles in farmland based on fusion of information from the mmWave radar and monocular camera. The method combines the advantages of the mmWave radar in range and speed measurement and the camera in type identification and lateral localization and uses the decision-level fusion algorithm of information and global nearest neighbor method for data association. The effective target sequence of the mmWave radar and the effective target sequence of camera with successful data association will be weighted and output, and the output information includes more accurate target orientation, longitudinal speed, and category. For the unassociated sequence, it is used as a new target and tracked by using the extended Kalman filter algorithm, and thus is processed and output according to the effective life cycle. 

The rest of the structure of this paper is as follows: Section 2 shows the related work about the fusion of the mmWave radar sensor and vision sensor for object detection. Section 3 introduces the materials and methods of this study, including the sensor space-time alignment and the fusion processing of the sensor information. Section 4 shows the experimental results of this study. Finally, the conclusions are drawn in Section 5.

## 2. Related Work

The fusion of the mmWave radar sensor and vision sensor is often used for road target detection. Huang et al. [16] proposed a simple method to fuse radar and vision sensor data to detect and track moving objects. The mmWave radar provided location information, while the vision sensor provided candidate regions in images. The invalid radar points were then filtered according to the speed information, which reduced the impact of stationary targets such as trees and bridges on the mmWave radar. Moreover, Wang et al. [17] presented a simple and feasible system scheme for road obstacle detection by using an mmWave radar and a monocular vision sensor. After radar-vision point alignment and region searching for potential target detection, obstacle detection was performed based on the adaptive threshold algorithm, and edge detection was used to assist in determining the boundary of the obstacles.

The above researches are based on data level fusion of millimeter wave radar and camera, and the fusion method used is relatively simple. However, some researches have developed in-depth data fusion algorithms. For example, Long et al. [18] proposed a sensor fusion system combining an RGB depth vision sensor and an mmWave radar sensor to realize obstacle detection for blind navigation. The position of obstacles was obtained by using the RGB depth sensor based on contour extraction and the MeanShift algorithm. The data fusion algorithm based on particle filter was used to fuse the RGB depth data and mmWave radar data to achieve accurate state estimation, while a collaborative fusion method between the mmWave radar and a monocular camera was proposed by Wang et al. [19] to achieve the balance between vehicle detection accuracy and computational efficiency. The mmWave radar detected the vehicles on the road and transmitted the position and size of the region of interest (ROI) to the image sequence from the monocular camera. Then, the visual processing module generated a square boundary in the image frame according to the transmitted ROI information and used the active contour method to detect vehicles within the square boundary. In addition, ref. [20] proposed a CameraRadarFusion-Net architecture by using the BlackIn training strategy to fuse the camera and radar sensor data of road vehicles. 

Data layer fusion can retain the information in the original data to the maximum extent, but because the number of original data are generally large and there are many noises, the amount of calculation is huge during fusion processing. Therefore, some other researchers use the complementarity on the features of the targets from the mmWave radar and camera. For example, Guo et al. [21] presented a method to detect pedestrians and obtain their dynamic information based on the fusion of the mmWave radar information and camera image information. First, the effective target signal was extracted from the original radar data through the intra frame clustering algorithm and the inter frame tracking algorithm. Then, the region of interest generation strategy and the improved fast target estimation algorithm were used to obtain more accurate potential target regions. Finally, the gradient histogram features of the potential area were extracted, and the support vector machine was used to judge whether it was a pedestrian. Moreover, a new spatial attention fusion obstacle detection method based on the mmWave radar and vision sensor was proposed for autonomous driving by Change et al. [22]. The proposed fusion method was embedded in the feature extraction stage, which leverages the features of the mmWave radar and vision sensor effectively. 

The feature layer fusion method is essentially a hypothesis verification method, mainly based on camera information, supplemented by the mmWave radar information. The mmWave radar information is used to quickly filter most of the target free areas to improve the detection efficiency. To ensure this advantage, it requires the high detection accuracy of the mmWave radar.

Compared with those resent studies, the main advantages of this paper are summarized as follows: 

(1) The fusion is used for obstacle detection of the multiple farmland targets, while it is mainly used for target detection in non-agricultural fields in the existing literature.

(2) Decision level data fusion is designed after the effective target detection of the mmWave radar and camera, which gives play to their respective advantages and improves the reliability and robustness of detection. The improved YOLOv5s algorithm is used for vision detection of farmland obstacles, as shown in [9]. 

(3) The global nearest neighbor method is used for data association during fusion. The uncorrelated sequence is tra cked as a new target using the extended Kalman filter algorithm, and then is processed and output during the effective life cycle.

## 3. Materials and Methods

### 3.1. Space-Time Reference Alignment of Sensors

#### 3.1.1. Time Reference Alignment

**Figure 2:**
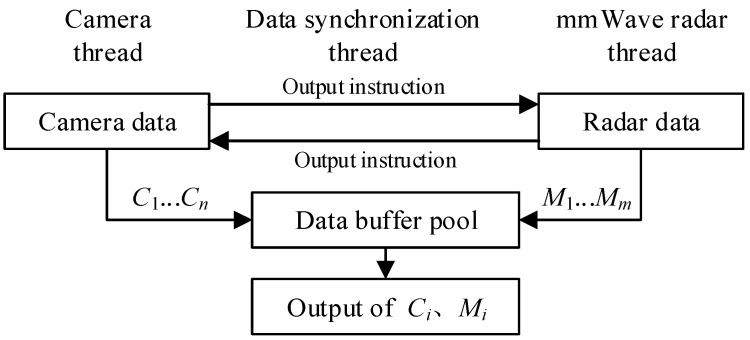
Process of time reference alignment between camera and mmWave radar.

Here the data acquisition synchronization thread was used to achieve time reference alignment. To achieve time synchronization, the camera thread, the mmWave radar thread, and the data synchronization thread were created in the program, as shown in Figure 2. The camera thread was used to receive and process the camera data *C*_1_, …, *C_n_*, and the mmWave radar thread was used to receive and process the radar data *M*_1_, …, *M_m_*. When the data synchronization thread was triggered, the camera data and the mmWave radar data at the same moment were output from the data buffer pool for future data fusion processing. 

#### 3.1.2. Transformation between mmWave Radar Coordinate System and Pixel Coordinate System

**Figure 3:**
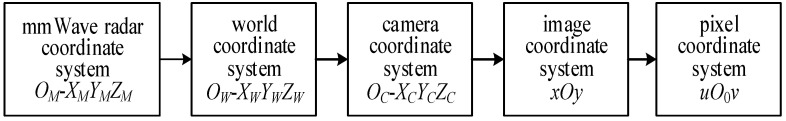
Transformation between different coordinate systems.

Here, the space reference alignment was realized through the transformation between the mmWave radar coordinate system and the pixel coordinate system. The transformation needed the assistance of some intermediate coordinate system, as shown in Figure 3.

**Figure 4:**
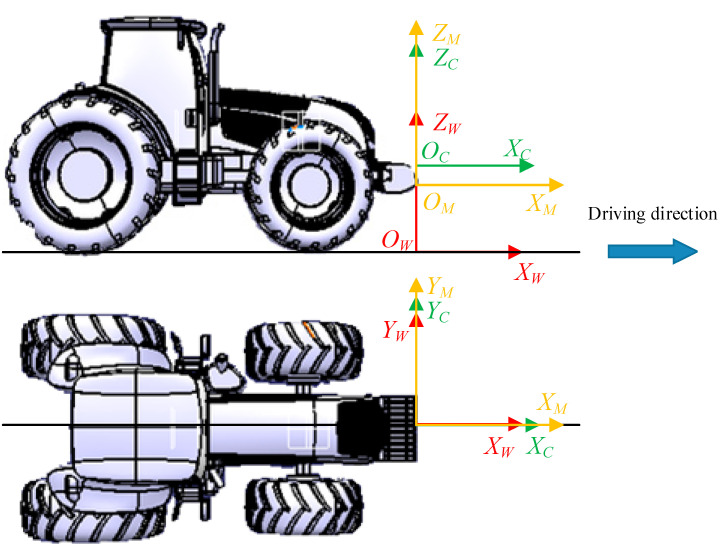
Relationship of the mmWave radar coordinate system, the camera coordinate system and the world coordinate system.

According to the actual installation position of the mmWave radar and the camera, the relationship between the mmWave radar coordinate system, the world coordinate system, and the camera coordinate system is shown in Figure 4. The *X_W_*-axis of the world coordinate system is consistent with the driving direction of the tractor, the *Z_W_*-axis is perpendicular to the ground upward, and the *Y_W_*-axis is perpendicular to the *X_W_O_W_Z_W_*-plane; the *Z_M_*-axis of the radar coordinate system and the *Z_C_*-axis of the camera coordinate system are perpendicular to the ground upward, the *X_M_O_M_Y_M_*-plane and the *X_C_O_C_Y_C_*-plane are parallel to the ground, and the *X_M_*-axis and the *X_C_*-axis are consistent with the driving direction of the tractor. 

In the world coordinate system, the coordinates of the mmWave radar target were expressed by Equation (1).

(1) $$\left[\begin{array}{c} X_{W}\\ Y_{W}\\ Z_{W}\\ 1 \end{array}\right]=\left[\begin{array}{c} X_{M}\\ Y_{M}\\ Z_{M}\\ 1 \end{array}\right]-\left[\begin{array}{c} 0\\ Ly\\ L_{Z}\\ 1 \end{array}\right]$$

where *L_y_* and *Lz* are the distances between the mmWave radar coordinate origin and the world coordinate system coordinate origin on the *Y*- and *Z*- axes, respectively.

**Figure 5:**
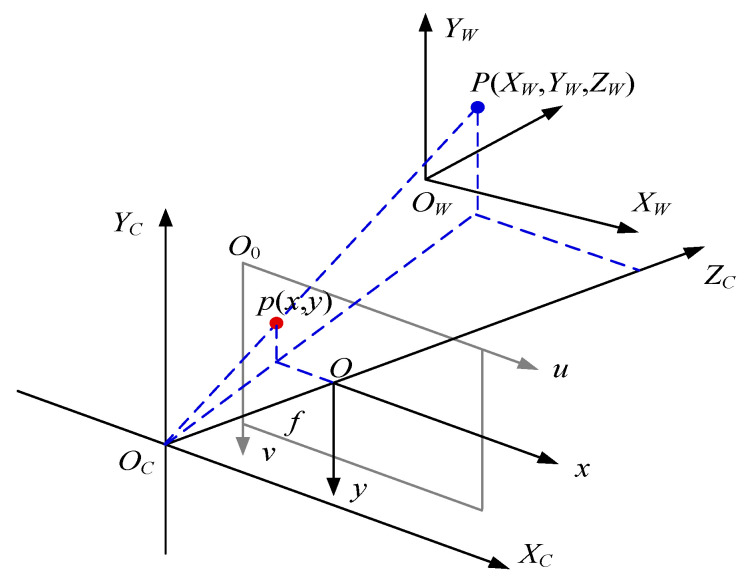
Transformation between the world coordinate system, the camera coordinate system, and the image coordinate system.

The transformation from the world coordinate system to the pixel coordinate system goes through the camera coordinate system and the image coordinate system, and the relationship between them is shown in Figure 5. The point *P*(*X_W_*, *Y_W_*, *Z_W_*) in the world coordinate system corresponds to the point *p*(*x*, *y*) in the image coordinate system. The *Z_C_*-axis of the camera coordinate system coincides with the *z*-axis of the image coordinate system, and the intersection *O* of the *Z_C_*-axis of the camera coordinate system and the imaging plane is used as the coordinate origin of the image coordinate system, and the plane formed by the *x*- and *y*-axes of the image coordinate system is parallel to the *X_C_O_C_Y_C_*-plane of the camera. The origin *O*_0_ of the pixel coordinate system *uO*_0_*v* is the upper left vertex of the imaging plane, the *u*-axis is parallel to the *x*-axis of the image coordinate system *xOy*, and the *v*-axis is parallel to the *y*-axis of the image coordinate system.

The transformation relationship from the world coordinate system to the pixel coordinate system is expressed in the following Equation (2).

$$Z_{C}\left[\begin{array}{l} u\\ v\\ 1 \end{array}\right]=\left[\begin{array}{ccc} \frac{1}{d_{x}} & 0 & u_{0}\\ 0 & \frac{1}{d_{y}} & v_{0}\\ 0 & 0 & 1 \end{array}\right]\left[\begin{array}{cccc} f & 0 & 0 & 0\\ 0 & f & 0 & 0\\ 0 & 0 & 1 & 0 \end{array}\right]\left[\begin{array}{cc} R & T\\ 0^{T} & 1 \end{array}\right]\left[\begin{array}{c} X_{W}\\ Y_{W}\\ Z_{W}\\ 1 \end{array}\right]$$

(2) $$=\left[\begin{array}{cccc} f_{x} & 0 & u_{0} & 0\\ 0 & f_{y} & v_{0} & 0\\ 0 & 0 & 1 & 0 \end{array}\right]\left[\begin{array}{cc} R & T\\ 0^{T} & 1 \end{array}\right]\left[\begin{array}{c} X_{W}\\ Y_{W}\\ Z_{W}\\ 1 \end{array}\right]=M_{2}M_{1}\left[\begin{array}{c} X_{W}\\ Y_{W}\\ Z_{W}\\ 1 \end{array}\right]$$

where *d_x_* is the dimension of each pixel point in the *x*-direction in the image coordinate system; *d_y_* is the dimension of each pixel point in the *y*-direction in the image coordinate system; *u*_0_ is the deviation from the camera optical axis to the center of the imaging plane in the *x*-direction; *v*_0_ is the deviation from the camera optical axis to the center of the imaging plane in the *y*-direction; *f* is the camera focal length; *R* is the rotation matrix; *T* is the translation matrix; 0*^T^* is the zero vector; *M*_2_ is the internal parameter matrix of the camera; *M*_1_ is the external parameter matrix of the camera.

### 3.2. Fusion Processing of Sensor Information

#### 3.2.1. Introduction to Obstacle Detection Algorithm

For the visual inspection module, this paper adopted improved YOLOv5s [9], automatically generated the anchor frame scale through the K-Means algorithm to speed up the convergence speed, and used the CIoU loss function to reduce false detection and missing detection to improve the accuracy. For the mmWave radar detection module, in order to reduce the amount of data during fusion input, a three-step filtering algorithm including empty target filtering, false target filtering, and non-threat target filtering was adopted, which was mainly realized through relative distance, effective target life cycle, and horizontal and vertical coordinate threshold. 

#### 3.2.2. Decision-Level Fusion of Sensor Information

**Figure 6:**
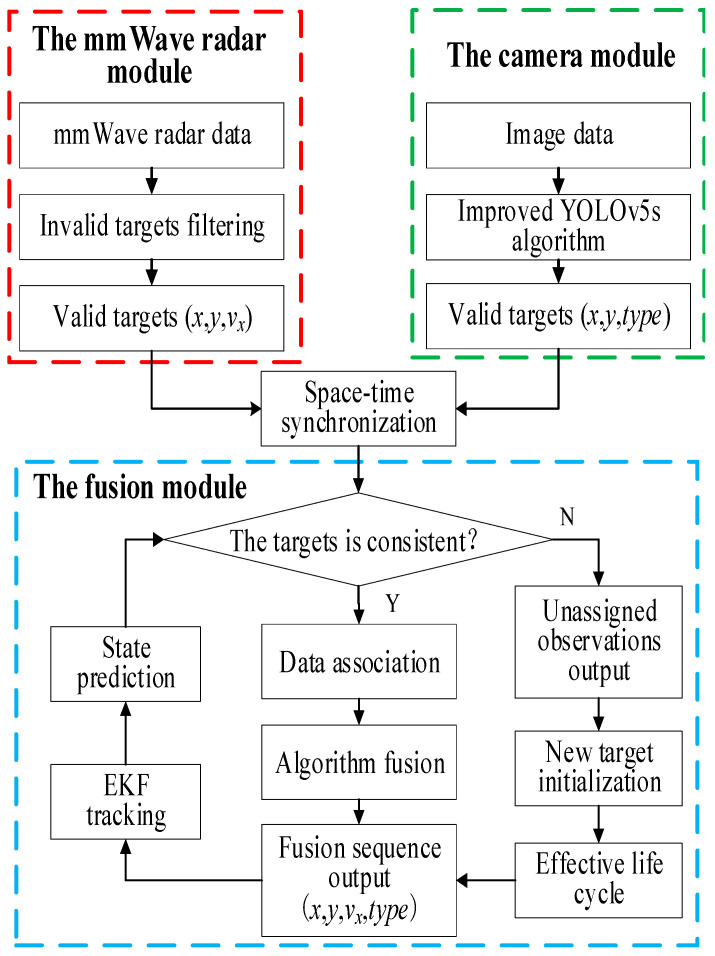
Framework of mmWave radar and camera information fusion.

The fusion framework divided into the mmWave radar module, the camera module, and the fusion module, as shown in Figure 6. In the mmWave radar module and the camera module, the mmWave radar and the camera performed target detection and output a sequence (*x*, *y*, *v_x_*) of a valid radar target and a sequence (*x*, *y*, *type*) of a valid visual target, respectively, where *x* and *y* were the values of the valid target in the world coordinate system, respectively, and *v_x_* and *type* were used as complementary information. 

Then, the valid targets detected by both sensors were output into the fusion module. The matching of the observations of the two sensors was first performed to determine which two observations were heterogeneous observations belonging to the same target. Using the state prediction value of the output sequence as a reference in this paper, the two heterogeneous observations that were most similar to this reference value were selected. If the two heterogeneous observations were similar to the reference value, data association was performed. Otherwise, the unmatched observations were initialized into a new target. The associated data were synthesized by the fusion algorithm, and thus the final target fusion sequence was output, which contained important information such as the target longitudinal coordinates, lateral coordinates, type, and velocity.

#### 3.2.3. Data Association Based on Global Nearest Neighbor Method

**Figure 7:**
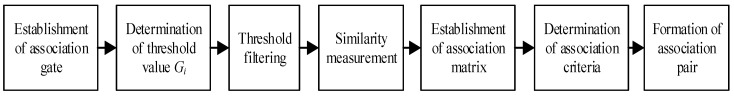
Steps of data association.

For the data association methods, there is mainly the global nearest neighbor (GNN) method, the probability data association (PDA) method, the joint probability data association (JPDA) method, and the multiple hypothesis tracking (MHT) method. Their advantages and disadvantages are shown in Table 1. Compared with the road environment, the farmland environment is an occasion with low target density, and generally the spacing between targets is large and the possibility of matching confusion is low. Therefore, the global nearest neighbor method was chosen due to less calculation and convenience in this paper. The method selected the association with the smallest total association distance, which effectively reduced the errors generated by the local nearest neighbor method during association. Figure 7 shows the steps of association between the camera observations and the mmWave radar observations.

(1) Establishment of association gate

**Figure 8:**
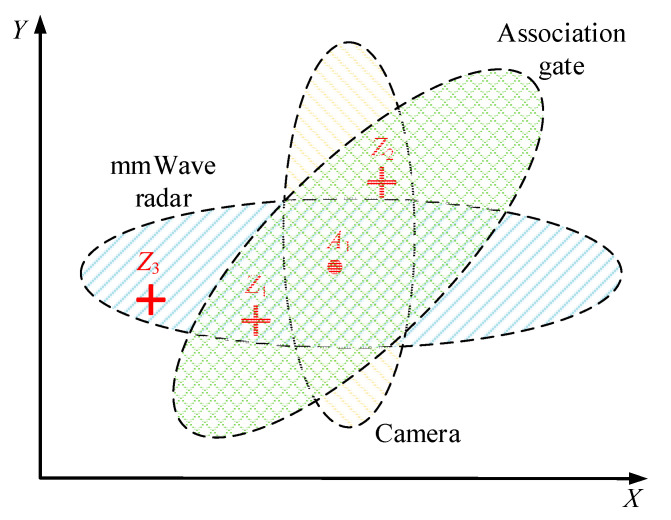
Setting up an association gate.

The purpose is to remove some observations that are far from the target, and to match only those within the association gate, which can reduce the computation of the subsequent association. The established association gate (Figure 8) integrated the longitudinal distance measurement error of the mmWave radar with the lateral distance measurement error of the camera.

In Figure 8, *A*_1_ (*X*_1_, *Y*_1_) is the predicted value of the target at the previous moment, with three nearby measurements *Z*_1_, *Z*_2_ and *Z*_3_. Assuming that the normalized distance of the observed values after the space–time calibration is defined as

(3) $$D^{2}=A^{T}S^{-1}A$$

where *A* is the observation error matrix; *S* is the error covariance matrix. 

The equation of the elliptic association gate is shown in the following equation:

(4) $$\frac{{\left(X-X_{1}\right)}^{2}}{{\left(k\sigma_{x}\right)}^{2}}+\frac{{\left(Y-Y_{1}\right)}^{2}}{{\left(k\sigma_{v}\right)}^{2}}=1$$

where *σ_x_*, *σ_y_* are the error covariances; *k* is a constant.

(2) Determination of threshold value *G_i_* and threshold filtering

*D*^2^ is a normalized random variable, and when the error between the observed and predicted values satisfies the normal distribution, *D*^2^ = *x* obeys the *χ*^2^ distribution with the degree of freedom *M*. Whether the observed values fall into the association gate becomes a statistical test problem. If *D*^2^ is less than the critical value *χ_α_*^2^, the statistical test is deemed to be accepted, as the following equation:

(5) $$f(x)=\frac{x^{\frac{1}{2}M-1}}{2^{\frac{1}{2}M}\Gamma \left(\frac{1}{2}M\right)}\exp\left(-\frac{1}{2}x\right)$$

where *M* is the dimension of observations and set *M* = 2 in this paper.

Then the probability of the observation falling into the association gate is calculated by the following equation:

(6) $$P=\int_{0}^{\chi_{\alpha }^{2}}f(x)dx$$

The bound of the association gate corresponds to *χ*^2^ and the size of the association gate is related to the error covariance. Therefore, the gate value *G_i_* was checked by the *χ*^2^ distribution table according to the degrees of freedom as well as the fall-in probability *P*.

(3) Similarity measurement

This step is used to measure the similarity between the observed and predicted values. In this paper, the distance between the observed and predicted values was calculated by using the Mahalanobis distance. The calculation of the Mahalanobis distance is shown below:

(7) $$d_{M}=\sqrt{{\left(Z_{i}-Z_{k/k-1}\right)}^{T}S_{k}^{-1}\left(Z_{i}-Z_{k/k-1}\right)}$$

where *S_k_*^−1^ the covariance matrix between the observed and predicted values.

(4) Establishment of the association matrix

Each different observation value and different predicted value were combined in pairs to calculate the Mahalanobis distance, and the calculated results were stored in a matrix, which was the association matrix.

(5) Determination of association criteria and formation of association pairs

In this paper, the global nearest neighbor method was used, whose core idea was to minimize the total association cost:

(8) $$\min\left\{\sum_{i=1}^{n}\sum_{j=1}^{n}C_{ij}x_{ij}\right\}$$

where *x_ij_* is a binary variable, zero means no association and 1 means association; *C_ij_* is the distance between measurement *i* and target *j*. When generating the matrix, each row and column only have one 1.

#### 3.2.4. Weighted Output of Observations 

Since a valid target only outputs a sequence containing its own information, the two observations needed to be weighted to output when the mmWave radar data were successfully associated with the camera data. Considering that the errors of distance detection of the mmWave radar was large in the lateral direction, while the errors of distance detection were large in the longitudinal directions, the respective errors of both sensors were suppressed through the weights by using the following equation:

(9) $$\left\{\begin{array}{l} x=\frac{x_{m}\mu_{cx}}{\mu_{mx}+\mu_{cx}}+\frac{x_{c}\mu_{mx}}{\mu_{mx}+\mu_{cx}}\\ y=\frac{y_{m}\mu_{cy}}{\mu_{mv}+\mu_{cv}}+\frac{y_{c}\mu_{my}}{\mu_{mv}+\mu_{cv}} \end{array}\right.$$

where, *x_m_*, *y_m_* are the observed values of the mmWave radar; *x_c_*, *y_c_* are the observed values of the camera; *μ_mx_*, *μ_my_* are the errors of the mmWave radar in *x*-axis and *y*-axis directions, respectively; *μ_cx_*, *μ_cy_* are the errors of camera in *x*-axis and *y*-axis directions, respectively.

The errors of the mmWave radar and the camera in the *x*-axis and *y*-axis directions satisfied the following conditions:

(10) $$\left\{\begin{array}{l} \mu_{mx}<\mu_{cx}\\ \mu_{cy}<\mu_{my} \end{array}\right.$$

#### 3.2.5. Target Tracking Based on Extended Kalman Filter 

Here, the extended Kalman filter (EKF) method was used for target tracking [23]. The state equation and the observation equation of the EKF are shown by the following equation:

(11) $$\left\{\begin{array}{l} X_{k}=f(X_{k-1})+W_{k}\\ Z_{k}=h(X_{k})+V_{k} \end{array}\right.$$

where *X_k_*, *X_k_*_−1_ are the state vectors of the target at moments *k*, *k −* 1; *Z_k_* is the observed vector of the target at moment *k*; *f*, *h* is the nonlinear state transfer matrix; *W_k_* is the process noise; *V_k_* is the observed noise. 

Thus, the time update equation of the EKF is as follows:

(12) $$\left\{\begin{array}{l} {\hat{X}}_{k|k-1}=f({\hat{X}}_{k-1|k-1})\\ {\hat{P}}_{k|k-1}=F_{k-1}P_{k-1|k-1}F_{k-1}^{T}+Q \end{array}\right.$$

where, ${\hat{X}}_{k\left|k-1\right.}$ is the state prediction value; ${\hat{P}}_{k\left|k-1\right.}$ is the prediction error covariance; *Q* is the covariance matrix of the process noise; *P_k−_*_1|*k−*1_ is the estimation error covariance; *F* is the Jacobi matrix of the state transfer matrix *f*. 

The observation update equation for the EKF is as follows:

(13) $$\left\{\begin{array}{l} K_{k}={\hat{P}}_{k|k-1}H_{k}^{T}{\left[H_{k}{\hat{P}}_{k|k-1}H_{k}^{T}+R\right]}^{-1}\\ {\hat{X}}_{k|k}={\hat{X}}_{k|k-1}+K_{k}[Z_{k}-h({\hat{X}}_{k|k-1})]\\ {\hat{P}}_{k|k}=(I-K_{k}H_{k}){\hat{P}}_{k|k-1} \end{array}\right.$$

where *K_k_* is the Kalman gain; *R* is the covariance matrix of the observed noise; *H* is the Jacobi matrix of the state transfer matrix *h*; ${\hat{X}}_{k\left|k\right.}$ is the updated target state estimation vector; ${\hat{P}}_{k\left|k\right.}$ is the updated error covariance; and *I* is the unit matrix. 

Under the discrete state model, the state vector and state transfer matrix of the fused target of this work were described as:

(14) $$x={\left[\begin{array}{llll} x & v_{x} & y & v_{y} \end{array}\right]}^{T}$$

(15) $$f=\left[\begin{array}{cccc} 1 & \Delta t & 0 & 0\\ 0 & 1 & 0 & 0\\ 0 & 0 & 1 & \Delta t\\ 0 & 0 & 0 & 1 \end{array}\right]$$

where *x*, *y* are the lateral and longitudinal coordinates of the target; *v_x_*, *v_y_* are the lateral and longitudinal velocities of the target.

The observed vector and the relative state transfer matrix are described as:

(16) $$z={\left[\begin{array}{ll} x & y \end{array}\right]}^{T}$$

(17) $$h=\left[\begin{array}{llll} 1 & 0 & 1 & 0\\ 0 & 0 & 0 & 0 \end{array}\right]$$

After the above series of calculations, the state vector evaluation of the target was obtained from the fused sequence that were output at the previous moment. Then the observation association was performed, and when some observations were not successfully matched, it was initialized to a new target that was tracked again by using the EKF. The life cycle theory was used to manage the valid target pool, that is, when the target appeared for three or more consecutive times, the target was output as a valid target; when the target was lost for five consecutive times, the target was removed.

## 4. Results and Discussion

### 4.1. Introduction to the Experimental Platform

**Figure 9:**
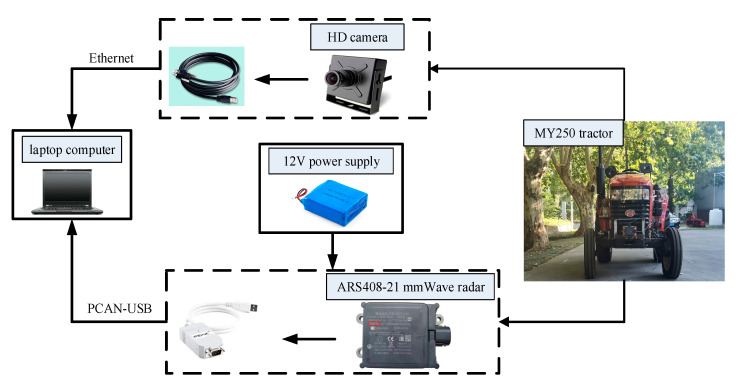
Experimental configuration.

An MY250 tractor was chosen as the experimental platform, and a ARS408-21 mmWave radar and an MCD-1073 HD camera were installed in front of it. A DELL lns15-7501 laptop computer was used to receive and process the radar information transmitted via a PCAN-USB and the camera information transmitted via Ethernet. The experimental configuration is shown in Figure 9.

### 4.2. Sensor Calibration 

The sensor calibration mainly included the internal and external parameters of the camera and the external parameters of the mmWave radar. The ultimate goal was to map the mmWave radar data points to the image through the space–time reference alignment. 

#### 4.2.1. Internal Parameter of Camera 

**Figure 10:**
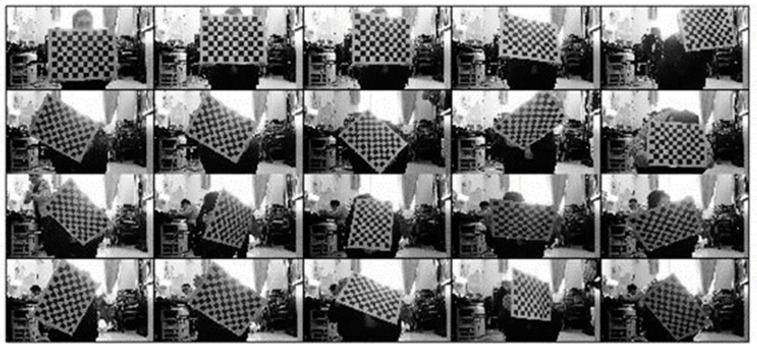
Checkerboard diagram from different perspectives.

Here Zhang’s calibration method was used to solve the internal parameters of the camera [24]. A checkerboard calibration board with 12 × 9 square grids was selected, with a single square size of 30 mm × 30 mm. The camera position was fixed and 20 images of the checkerboard with different angles and distances were taken, as shown in Figure 10. 

The toolbox toolbox_calib based on MATLAB was used to solve the internal parameters of the camera. The above 20 images were input and the calibration tool automatically extracted the checkerboard grid corner points of the selected area. The final internal parameter matrix of the camera was obtained as:

(18) $$M_{2}=\left[\begin{array}{cccc} 1977.54 & 0 & 1033.62 & 0\\ 0 & 2011.78 & 700.10 & 0\\ 0 & 0 & 1 & 0 \end{array}\right]$$

#### 4.2.2. External Parameters of mmWave Radar and Camera

The external parameters of the mmWave radar and the camera are related to their installation position, which mainly includes the translation and rotation vectors. The installation plane of the mmWave radar and the camera is perpendicular to the ground and in the same installation plane (see Figure 9). The vertical distance between the camera’s coordinate origin and the ground is 1.33 m, so the camera’s translation vector is T = [0 0 1.33], and the vertical distance between the coordinate origin of the mmWave radar and the ground is 1.24 m, so *L_z_* = 1.24. In addition, the mmWave radar and the camera are mounted on the centerline of the tractor, so *L_y_* = 0.

#### 4.2.3. Solving of Pixel Value-Vertical Distance Relationship Function 

**Figure 11:**
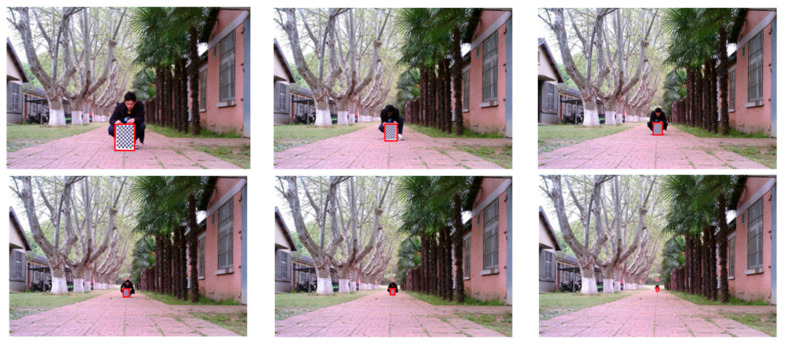
Calibration plate position.

At the beginning, the calibration plate was placed 4.8 m directly in front of the tractor, and the calibration was moved every 2.4 m and photographed. A total of 22 images were taken in total to fit distances in the range from 4.8 to 55.2 m. Some of the images are shown in Figure 11 below. 

**Figure 12:**
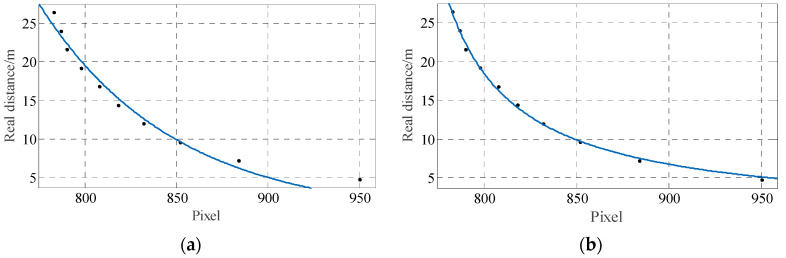

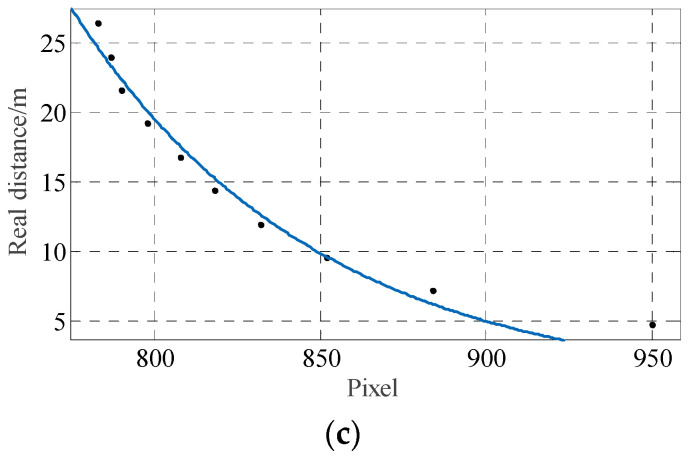
Fitting curves. (**a**) Power function; (**b**) Rational function; (**c**) Exponential function.

By using the curve fitting toolbox of MATLAB, three group of curves with good fitting effects, such as power function, rational function, and exponential function, were obtained by using different fitting functions for 22 sets of data, as shown in Figure 12. 

The performance of the fitted curves was evaluated by three parameters, such as the sum of squared errors (SSE), root mean squared error (RMSE), and coefficient of determination (R-square). The closer the SSE is to 0, the closer the RMSE is to 0, and the closer the R-square is to 1, the better the selected curve fits the data. The three evaluation parameters of the above three fitted curves are shown in Table 2.

From Table 2, all three parameters of the rational function are optimal; therefore, the rational function is chosen as a function of the pixel value versus the distance, as shown in the following equation:

(19) $$d=\frac{998.6}{v+747.2}$$

where *d* is the distance between the target and the camera; *v* is the longitudinal pixel coordinate value of the midpoint of the bottom edge of the target bounding box. 

### 4.3. mmWave Radar and Camera Information Fusion Test

The methods mentioned in this paper were implemented in the ROS environment of Ubuntu 16.04. During the tests, the radar data frames and image frames were captured every 120 ms for information fusion, and the radar data were visualized using the Rviz 3D platform based on ROS. 

**Figure 13:**
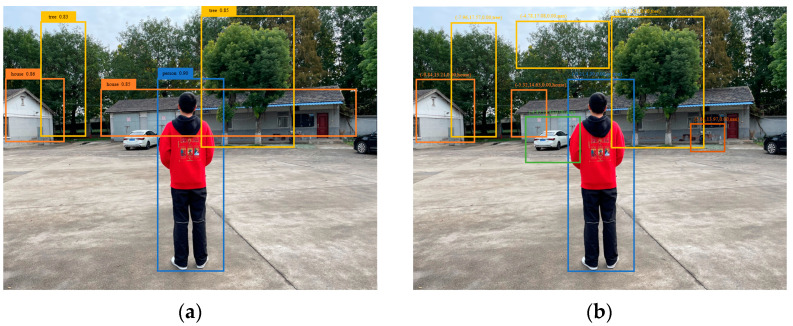
Information fusion detection test of mmWave radar and camera in non-agricultural environment. (**a**) Detect with camera only. (**b**) Fusion detection of camera and mmWave radar.

Firstly, the effect of the information fusion of the mmWave radar and the camera was tested in a non-agricultural environment, as shown in Figure 13. In Figure 13a, three obstacle targets such as people, houses, and trees were detected with the camera only, by using the improved YOLOv5s algorithm in the literature [9]. But after the data fusion, 8 targets sequences including position, longitudinal speed, and category were output: (−9.84, 13.21, 0.00, house), (−7.96, 17.57, 0.00, tree), (−5.32, 14.63, 0.00, house), (−0.21, 4.30, 0.00, person), (4.36, 13.51, 0.00, tree), (−4.78, 17.08, 0.00, nan), (−4.85, 12.83, 0.00, nan), (5.64, 13.97, 0.00, nan), as shown in Figure 13b.

In Figure 13, both the mmWave radar and the camera detected the people, houses, and trees, but cars and electric motorcycle cars were not detected by the cameras because they were not regarded as farmland obstacles trained in the dataset, while these targets were detected by the mmWave radar and the output target sequences. 

Then, we captured 500 images frames and the mmWave radar data frames at the same sampling period in the farmland environment. There were 953 targets in the 500 images, and the target categories and quantities are shown in Table 3, where the other obstacles referred to those that would pose a threat to the farm machinery, but did not belong to trees, people, tractors, haystacks, houses, wire poles, and sheep. 

**Figure 14:**
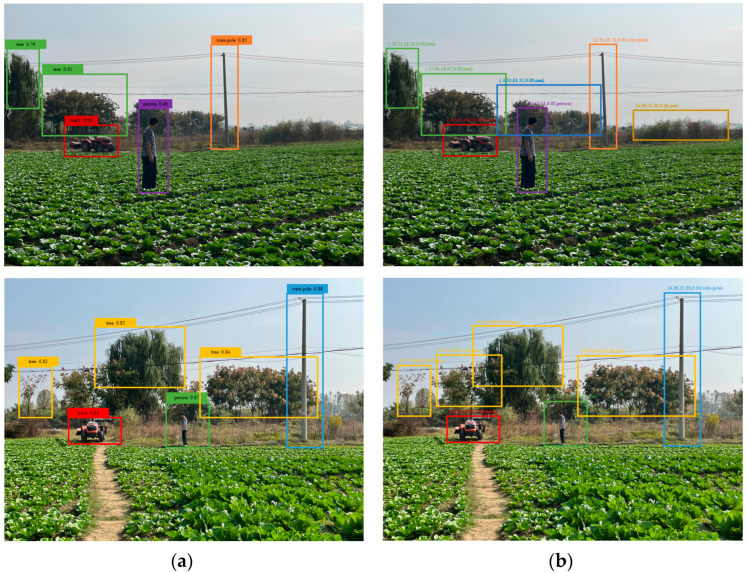
Detection tests in farmland environment. (**a**) Camera-only detection. (**b**) Fusion detection.

The 953 targets were tested in the two methods: the camera-only detection and the fusion detection. Sometimes farmland obstacles were missed by the camera-only detection due to occlusion or implicit features, but the number of undetected obstacles were significantly reduced by using the fusion detection, as shown in Figure 14. The test results are shown in Table 4. From Table 4, the fusion detection method has better detection with the average accuracy rate of a target detection of 86.18%, which is higher than that of the camera-only detection method with 62.47%. The average missing rate by adding the mmWave radar is reduced by 13.71% compared to that of the camera-only detection method. 

The results of the camera-only detection and fusion detection for various types of obstacles are shown in Table 5. Compared with the camera-only detection method, the fusion detection method improves the accuracy of different obstacles, especially the detection accuracy of human and tractor obstacles is increased by 12.12% and 23.81%, respectively.

### 4.4. Comparison between This Study and Other Sensor Fusion Algorithms 

At present, there are three kinds of sensors fusion algorithms: data level, feature level and decision level. Therefore, a contrast experiment was set based on the same obstacle (person) in the same environment. The experimental results are shown in Table 6. From Table 6, the detection accuracy of the decision level fusion algorithm used in this paper reaches 95.19%, which was superior to 90.81% of the feature level fusion and 88.56% of the data level fusion. The detection accuracy of all three was relatively high due to the relative simplicity of the obstacles used in the contrast test. However, the decision level fusion method used in this paper had the highest accuracy because the program had processed the data of each sensor before the fusion detection, which reduces the human impact.

## 5. Conclusions

A method for detecting obstacles in farmland based on the information fusion of a mmWave radar and a camera was proposed to remedy the defects of single sensor in robustness, accuracy, and redundancy of target detection. The method combined the advantages of the mmWave radar in range and speed measurements and the camera in type identification and lateral localization and used decision-level fusion as well as the global nearest neighbor method for data association. The valid target sequences from the mmWave radar and the camera were weighted to output after successful data association, and the output information included more target orientation, longitudinal velocity, and category. For the unassociated sequences, they were tracked as new targets using the EKF algorithm and were processed and output during the effective life cycle of the targets.

To verify the effectiveness of the proposed method, an experimental platform was built based on a tractor, and then the external parameters of the mmWave radar and the internal and external parameters of the camera were solved. The experiments were conducted in the ROS environment for real farmland obstacle detection. The results of the experiments show that the proposed method has better detection with the average accuracy rate of target detection of 86.18%, which was higher than the average accuracy rate of target detection of 62.47% for the camera-only detection method. The average missing rate by adding the mmWave radar was reduced by 13.71% compared to that of the camera-only detection method. Moreover, the accuracy rate by using the fusion detection increased for the different obstacles compared with the camera-only detection method. Furthermore, through the contrast experiment of the sensor fusion algorithms, the detection accuracy of the decision level fusion algorithm used reaches 95.19%, which was superior to 90.81% of the feature level fusion and 88.56% of the data level fusion. This will lay a solid foundation for the follow-up research on obstacle avoidance of unmanned agricultural machinery. In addition, due to the insufficient dataset and relatively simple experimental environment in this paper, future studies will need to focus on expanding the dataset and conducting experiments in rainy days or strong light conditions.

## Figures and Tables

**Table 1:** Advantages and disadvantages of data association methods.

Methods	Advantages	Disadvantages	Degree of Difficulty	Scope of Application
GNN	Calculation is small and simple	When the target density is large, association errors are likely to occur	Easily	Target density is small
PDA	Applicable to target tracking in clutter environment	Difficult to meet real-time requirements	Difficult	Target tracking in clutter environment
JPDA	Better adapt to target tracking in dense environment	Phenomenon of combined explosion of calculated load may occur	Easily	Tracking of dense maneuvering targets
MHT	Better adapt to target tracking in dense environment	Too much prior knowledge depending on target and clutter	Difficult	Tracking of dense maneuvering targets

**Table 2:** The performance comparison of the three fitting curves.

	SSE	R-Square	RMSE
Power function	209.4915	0.9589	3.2364
Rational functions	33.9776	0.9933	1.3034
Exponential functions	227.8865	0.9553	3.3755

**Table 3:** Target categories and quantities.

	Tree	Human	Tractor	Haystack	House	Wire Poles	Sheep	Other
Number	175	154	203	62	166	94	57	42

**Table 4:** Results of the comparison of camera-only detection and fusion detection.

Method	Average Rate of Accuracy Detection (%)	Average Rate of Missing Detection (%)
Camera-only detection	62.47	27.51
Fusion detection	86.18	13.80

**Table 5:** Comparison of single sensor and multi-sensor detection results on all kinds of obstacles.

Category	Accuracy of Camera-Only Detection (%)	Accuracy of Fusion Detection (%)
Human	83.07	95.19
Tractors	73.09	96.90
Sheep	55.60	61.06
Haystack	51.66	66.32
Wire poles	60.01	73.11
House	42.24	53.78
Trees	40.81	48.02
Other	0.00	40.00

**Table 6:** Comparison of sensor fusion algorithms.

Integration Mode	Accuracy (%)
Data level fusion	88.56
Feature level fusion	90.81
Decision level fusion (in this paper)	95.19

## Data Availability

Raw data supporting the conclusions of this article will be made available by the authors upon requests for research purposes.
